# Clinical Presentation and Diagnostic Challenges of Congenital Thoracoabdominal Wall Defects in Dogs: Insights from a Case Series and Literature Synthesis

**DOI:** 10.3390/ani16050701

**Published:** 2026-02-24

**Authors:** José M. Cozar, Luis Avedillo, Nieves Martín-Alguacil

**Affiliations:** Research Group GIMCAD 971005-UCM, Departmental Section of Anatomy and Embryology, School of Veterinary Medicine, Universidad Complutense de Madrid, 28040 Madrid, Spain; jcozar@ucm.es (J.M.C.); luiavedi@ucm.es (L.A.)

**Keywords:** body wall defects, ectopia cordis, peritoneopericardial hernia, phenotypic overlap, Sternal–Body Wall Complex (STBWC), Sternal–Limb–Body Wall Complex (STLBWC), thoracoabdominoschisis, thoracoschisis, umbilical cord malformations

## Abstract

Congenital thoracoabdominal wall defects in dogs are uncommon and challenging to classify due to their overlapping anatomical and developmental features. This study analyzes three original canine cases alongside 17 published cases to clarify the relationships among Cantrell syndrome (CS), amniotic band syndrome (ABS), and body stalk anomaly (BSA). All of the original cases exhibited thoracoabdominal involvement with variations in umbilical cord morphology and associated anomalies. A comparative analysis revealed that these conditions form a syndromic continuum rather than distinct entities, influenced by the timing and mechanism of embryonic disruption. Early developmental insults were associated with multisystem malformations resembling CS or BSA, whereas later vascular disruptions produced more localized defects, such as gastroschisis. Umbilical cord morphology emerged as a key diagnostic discriminator across cases. Based on these findings, we developed an anatomically driven diagnostic decision tree to support clinical evaluation when information is incomplete. This study emphasizes the importance of integrating embryologic context with anatomical assessment and identifies significant gaps in molecular and genetic data. A developmental continuum model offers a more flexible, clinically meaningful framework for diagnosing congenital body wall defects in dogs.

## Simple Summary

Congenital defects of the thoracic and abdominal body wall are rare in dogs, yet they can be severe and difficult to classify. Veterinarians often use human medical terminology, such as Cantrell syndrome, amniotic band syndrome, and body stalk anomaly, to describe these conditions. However, many affected animals exhibit features that overlap with more than one syndrome. In this study, we examined three new cases of canine body wall defects and reviewed 17 published cases to better understand how these defects develop and how they should be diagnosed. Our findings suggest that these syndromes form a continuum rather than distinct categories. The timing of embryonic disruption and the appearance of the umbilical cord are especially important for distinguishing syndromic from nonsyndromic defects. Based on these insights, we created a practical diagnostic decision tree to help clinicians evaluate affected neonates, even when only partial information is available. Our research underscores the necessity of clearer diagnostic guidelines and enhanced documentation of congenital body wall defects in veterinary medicine.

## 1. Introduction

Although complex congenital body wall anomalies are rare in small animal practice, they represent an important diagnostic challenge for veterinarians due to their severity, variable presentation, and frequent association with life-limiting defects [1,2,3]. Cantrell syndrome (CS), body stalk anomaly (BSA), and amniotic band syndrome (ABS) are of particular clinical interest because they present with overlapping thoracoabdominal defects that complicate case assessment and decision-making [4,5,6]. Although these syndromes are well documented in human medicine, there are few reports in dogs, which often results in diagnostic uncertainty and limited guidance for clinicians managing affected neonates [2].

CS, also known as pentalogy of Cantrell (PC), is characterized by a group of midline defects affecting the diaphragm, abdominal wall, pericardium, heart, and sternum [7]. In veterinary patients, CS usually presents as thoracoabdominoschisis (ThAb) without associated spinal or limb abnormalities [4]. A defining feature is ectopia cordis (EC), wherein the heart is partially or fully outside the thoracic cavity. EC can occur in several anatomical forms, with the thoracoabdominal type being the most common form associated with PC [8]. EC has been documented in multiple domestic species, including cattle [9,10,11,12,13,14], cats [15], sheep [16], pigs [17,18,19,20,21,22,23,24], and dogs [25,26], but true EC remains exceptionally rare. The condition is believed to arise from defects in early cranial folding or ventral mesoderm development [7], though its etiology is likely multifactorial [27]. Clinically, EC may coexist with diaphragmatic defects, sternal malformations, and abdominal wall anomalies, such as omphalocele, abdominoschisis (Ab), or gastroschisis [28]. Variability in umbilical cord (UC) structure and insertion complicates classification and may influence prognosis. This study addresses the key clinical question of whether all such combinations fall within the spectrum of PC or if certain presentations, such as gastroschisis or diaphragmatic herniation without sternal involvement, represent distinct entities.

BSA is a severe congenital condition characterized by failure of ventral body wall closure, skeletal abnormalities, and UC malformations or absence. If limb defects are also present, the condition is termed Limb Body Wall Complex (LBWC) [29]. In this study, LBWC is treated as a BSA subtype, reflecting its hierarchical relationship rather than diagnostic inconsistency [1,5]. BSA is believed to result from the disruption of early embryonic folding and mesodermal fusion. Although BSA is well described in human fetopathology, reports in veterinary medicine, particularly in dogs and cats, remain limited [2,3]. A classification system originally developed in porcine models has proven useful for veterinary cases [1]. It helps clinicians distinguish between thoracoabdominal and abdominal defects, as well as between structural and nonstructural skeletal anomalies [30]. This framework is valuable for evaluating neonates with extensive malformations.

Unlike CS and BSA, ABS arises from an extrinsic mechanical process. In this process, fibrous amniotic strands can entangle or constrict fetal tissues. This leads to defects such as limb amputations, craniofacial abnormalities, and body wall disruptions [6,31,32,33,34,35,36,37]. ABS is usually sporadic and nonheritable [38], but it has been linked to asymmetric malformations and thoracoabdominal defects in dogs [2]. The critical window for band formation in dogs and cats is estimated to occur between days 10 and 20 of gestation [39,40]. Diagnosis is often based on characteristic lesions, even when bands are not visibly present. Differentiating ABS from amniotic sheets, which are non-adherent membranes that do not cause malformations, is essential for accurate case interpretation [6].

The terminology used to describe these defects is inconsistent in the veterinary literature. Overlapping terms, such as ABS, amniotic band sequence, amniotic disruption complex, LBWC, and ADAM sequence, are applied variably [2,3,29,41,42,43,44]. ABS generally refers to anomalies caused by fibrous amniotic bands. In contrast, “amniotic band sequence” emphasizes the cascade of developmental disruptions following amnion rupture [42,45]. Both are encompassed within the broader amniotic disruption complex (ADC), whose pathogenesis is still debated. The extrinsic theory, proposed by Torpin (1965) [46], attributes these defects to early amnion rupture and subsequent fetal entanglement [47], while the intrinsic theory proposes a vascular origin involving endothelial injury and secondary tissue necrosis [48]. Understanding these mechanisms is clinically relevant because the timing and nature of the insult influence the pattern and severity of malformations.

This study examines CS, BSA, and ABS, which together represent a spectrum of congenital thoracoabdominal defects with distinct pathophysiological mechanisms ranging from mesodermal developmental failure to mechanical entrapment. Distinguishing among these conditions is essential for veterinarians to make accurate diagnoses and prognoses and to communicate effectively with breeders and owners. Recent human studies have emphasized that these syndromes frequently overlap and may reflect a shared developmental continuum rather than distinct entities. This highlights the importance of comparative approaches when evaluating complex ventral body wall defects. To contextualize the canine findings within this broader developmental framework, the present study integrates three original cases with 17 additional cases identified through a structured literature review. Including these published cases was necessary to capture the full range of phenotypic variation, address the rarity of these defects in dogs, and enable meaningful comparisons with emerging human data [49]. By combining new clinical observations with comparative developmental insights, this study aims to clarify the relationships among CS, BSA, and ABS in dogs and support a more consistent, anatomically grounded classification of complex congenital malformations in veterinary medicine.

## 2. Diagnostic Framework for Classifying Thoracic and Abdominal Wall Malformations

### 2.1. Diagnostic Criteria for BSA

The cases were classified under BSA according to the diagnostic criteria established by Martín-Alguacil and colleagues using porcine and human models [1,5,29]. Although the system was originally developed for pigs, it is based on fundamental anatomical and embryological principles—embryonic folding, ventral body wall formation, and skeletal development—that are conserved across mammals. For this reason, the classification can be applied to canine congenital malformations. The BSA system has been applied consistently to hundreds of documented cases across multiple species, including pigs, humans, and other animals, demonstrating strong reproducibility [1,2,3,5,29]. The system relies on objective macroscopic criteria, particularly skeletal anomalies of the limbs, spine, and sternum, rather than subjective interpretation. Therefore, formal inter-rater agreement testing was not performed. The system distinguishes eight types of BSA. Types I–IV involve abdominal schisis (Ab) with varying combinations of limb and spinal anomalies. Types V and VI involve thoracic abdominal schisis (ThAb) with sternal and/or spinal anomalies. Types VII–VIII involve Ab with sternal and spinal anomalies or spinal anomalies alone. Complementary classifications were also applied to describe the relationship between body wall defects and skeletal anomalies: These include the Spinal Body Wall Complex (SPBWC), Spinal Limb Body Wall Complex (SPLBWC), Sternal Body Wall Complex (STBWC), and Sternal Spinal Body Wall Complex (SSBWC) [5].

### 2.2. Diagnostic Criteria for CS

CS is classified into three categories based on the presence and combination of five characteristic anomalies: (1) Midline supraumbilical abdominal wall defect; (2) lower sternal defect; (3) deficiency of the anterior diaphragm; (4) defect in the diaphragmatic pericardium; and (5) intracardiac anomalies [8].

Class 1 (complete form) includes all five defects. Class 2 (probable form) includes four defects and requires both intracardiac and abdominal wall anomalies. Class 3 (incomplete form) includes variable combinations that do not meet the full criteria. Originally proposed by Toyama in 1972 [50], this classification remains the standard for clinical and pathological diagnosis. Congenital peritoneopericardial hernia is not one of the five classic CS anomalies, but it may fall within Class 3 when it occurs alongside other body wall defects. This interpretation aligns with Toyama’s framework, which allows for broader phenotypic variability in incomplete forms [8].

### 2.3. Anatomical Classification of Body Wall Defects

Body wall defects were classified according to their anatomical location, structural characteristics, and associated anomalies. Omphalocele is a supraumbilical midline defect with herniated viscera enclosed in an amnion-peritoneum sac [2]. The term Ab was described as a large midline defect without a protective sac. Gastroschisis is a right-lateral defect adjacent to the umbilicus that is typically uncovered [51]. ThAb is a continuous defect that extends from the thorax to the abdomen and is often associated with ectopia cordis (EC) [2]. Each condition was evaluated in relation to its embryological origin and phenotypic overlap with PC and BSA. A Venn diagram was used to visualize the relationship between body wall defects and sternal anomalies, mapping each dog according to defect location and extent. The critical period of canine ventral body wall closure (gestational days 20–30) guided the embryological interpretation.

**Figure 1:**
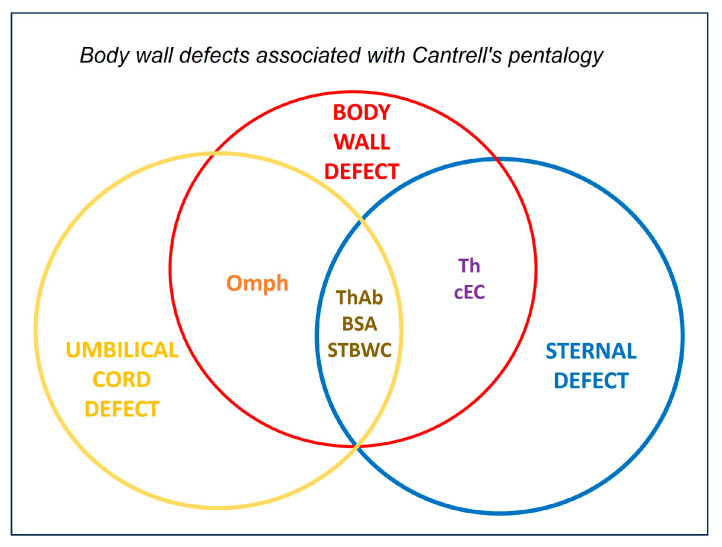
Anatomical relationship between body wall and sternal defects. Venn diagram illustrating the anatomical and developmental overlap between body wall defects (red circle) and sternal defects (blue circle). Th is positioned within both domains, reflecting its dual involvement. BSA and STBWC occupy the intersection, representing syndromic conditions with combined thoracic and abdominal wall failure. This framework supports the classification of the three dog cases, all presenting Th, within the overlapping region, with variations in abdominal defect location and UC involvement. BSA, Body Stalk Anomaly; cEC, celomic *Ectopia cordis*; Omph, omphalocele; STBWC, Sternal Body Wall Complex; Th, Thoracoschisis; ThAb, Thoracoabdominoschisis.

A Venn diagram was used to develop an anatomical classification framework that visualized the relationship between body wall defects and sternal defects (Figure 1). Representative conditions included omphalocele, Ab, ThAb and structural sternal defects. Thoracoschisis (Th) was positioned within both domains to reflect its dual involvement. Each dog case was mapped onto the diagram according to the location and extent of the defects. Embryological interpretation was guided by the known timeline of canine gestation, with a focus on the critical period of ventral body wall closure (gestational days 20–30). The anatomical findings were correlated with developmental timing to infer the origin and severity of each defect.

### 2.4. Classification Scheme for EC

Four types of EC were considered, based on associated anatomical defects. Type 1: Diaphragmatic defect with omphalocele/abdominal wall defect (Ab) and anomalous umbilical cord (UC). Type 2: Sternocostal defect (thoracoschisis) with omphalocele/abdominal wall (Ab) defect and anomalous umbilical cord (UC). Type 3: Sternocostal defect with a supraumbilical abdominal wall (Ab) defect due to rectus abdominis diastasis and a normal umbilical cord (UC). Type 4: Sternocostal defect with gastroschisis and normal UC [8].

### 2.5. Literature Review and Case Selection Criteria

A comprehensive literature review was conducted using Scopus, PubMed, Web of Science, and Google Scholar. The keywords used were “body wall defects,” “body stalk anomaly,” “ectopia cordis,” “pentalogy of Cantrell,” “Cantrell syndrome,” “peritoneopericardial diaphragmatic hernia,” and “amniotic band syndrome.” Additional searches targeted canine congenital malformations. To ensure accurate morphological characterization, only individuals with complete clinical records, diagnostic imaging, and/or necropsy documentation were included. Records lacking essential diagnostic information were excluded. Three previously published cases of canine peritoneopericardial hernias were not considered because the dogs did not exhibit the combination of sternal defects or intracardiac anomalies required for inclusion. Additionally, the original authors did not classify these cases as compatible with Cantrell syndrome [52,53,54]. A comparative analysis of published diagnoses and the anomalies observed in the present cases enabled the refinement of diagnostic categories.

## 3. Case Series Description

This case series includes three dogs with congenital thoracoabdominal body wall defects, with or without associated sternal, pericardial, or diaphragmatic anomalies. The three dogs examined exhibited ventral body wall closure defects with externalized thoracic and abdominal viscera. All specimens underwent detailed gross anatomical evaluation, complemented by advanced imaging modalities, including computed tomography (CT), three-dimensional reconstructions, and magnetic resonance imaging (MRI), depending on the availability of equipment at the time of presentation. The main characteristics of the cases are summarized in Table 1.

All specimens were obtained and examined in accordance with European Union Directive 2010/63/EEC and Spanish legislation RD 53/2013. Examinations were conducted by the GIMCAD 971005-UCM research group at the Universidad Complutense de Madrid, which is dedicated to studying congenital malformations in domestic animals. The terminology follows overlapping diagnostic frameworks, and each dog is described under multiple systems when phenotypic features coincide. This reflects the continuum of congenital thoracoabdominal anomalies. Due to the condition of the specimens and imaging constraints, not all malformations could be quantified. Therefore, qualitative descriptions were provided when measurements were not feasible. Gross dissection confirmed structural differences, and CT and MRI reconstructions provided three-dimensional visualization of the defects.

### 3.1. Case 1

**Figure 2:**
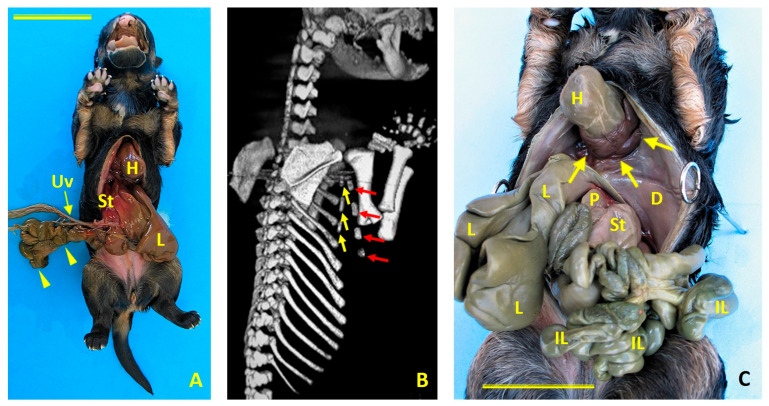
Congenital Body Wall Defects in Case 1 (Female German Shepherd). (**A**) Ventral view showing the thoracoabdominoschisis (ThAb) with externalized viscera. H: heart; L: liver; St: stomach; Uv: umbilical vein. Scale bar: 5 cm. (**B**) Computed tomography view of the thoracic and cervical malformations. Right dorsolateral view showing a bifid sternum. The yellow arrows indicate the three left sternites, and the red arrows indicate the four right sternites. (**C**) Ventral view. A ventral diaphragmatic defect (indicated by the arrows) allows the heart (H) to protrude externally without parietal pericardial coverage, consistent with cardiac ectopia. The diaphragm (D), intestinal loops (IL), liver (L), pancreas (P), and stomach (St) are also visible through the thoracoabdominal defect. Scale bar: 3 cm.

Case 1 was a female German shepherd with thoracoabdominoschisis (ThAb) and an abnormal umbilical cord (UC) (Figure 2A). The dog was scanned using the MultiVET multi-modality system equipped with a 32 kW X-ray generator and a 100 µm high-resolution flat panel detector. This system was manufactured by SEDECAL (Spanish Society of Electromedicine and Quality, S.A.) in Algete, Madrid, Spain. Gross and imaging findings revealed a short cleft sternum (Figure 2B), an absent ventral diaphragm (Figure 2C), and a complete absence of the pericardium.

**Figure 3:**
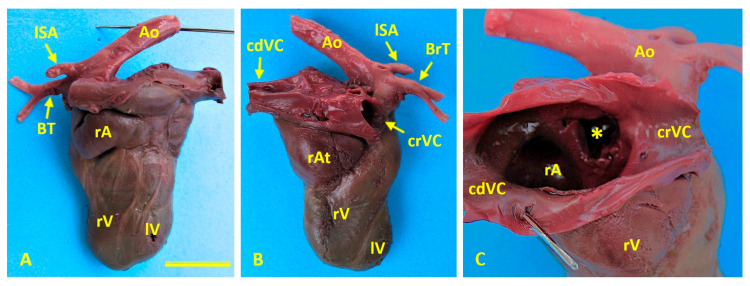
Cardiac morphology in Case 1 (female German Shepherd). (**A**) Left view showing an enlarged right auricle (rA) and hypoplastic left ventricle (lV). (**B**) Right view showing marked dilatation of the right atrium (rAt) and hypoplasia of the left ventricle (lV). (**C**) An internal view of the right atrium (rAt) reveals a prominent interatrial defect (asterisk), which creates direct communication between the atria. Ao: aorta; BrT: brachiocephalic trunk; cdVC: caudal vena cava; crVC: cranial vena cava; lSA: left subclavian artery; rV: right ventricle.

Cardiac anomalies included ectopia cordis, mitral valve stenosis, tricuspid valve dysplasia, hypoplasia of the left ventricle, marked dilation of the right atrium, and an atrial septal defect that created direct interatrial communication (Figure 3).

**Figure 4:**
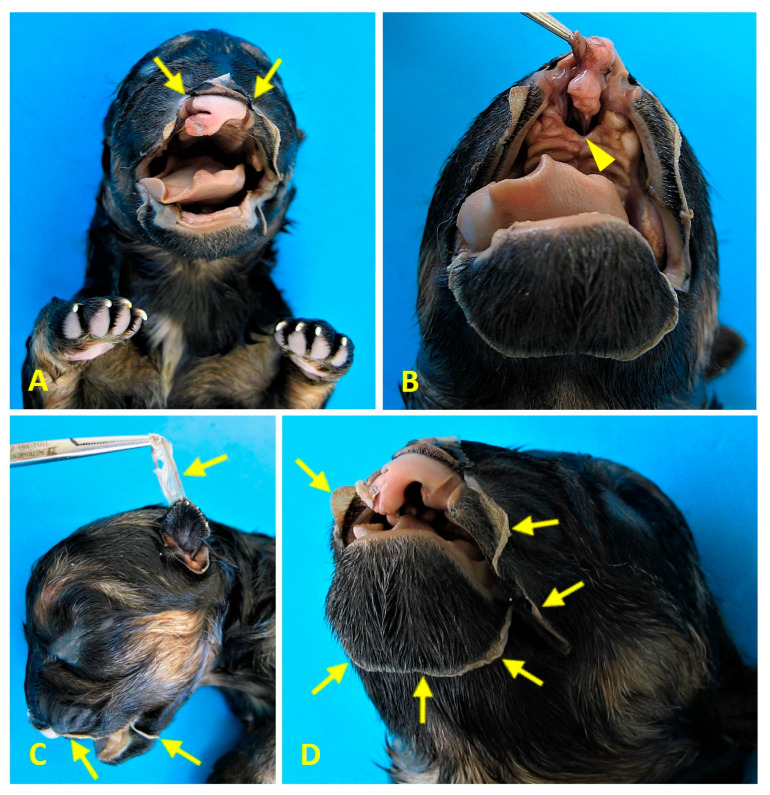
Case 1 (female German shepherd): Craniofacial anomalies (**A**,**B**) and cranial amniotic adhesions (**C**,**D**). (**A**) Rostral view showing bilateral cheiloschisis (arrows), affecting both sides of the upper lip. (**B**) A ventrorostral view of the roof of the oral cavity shows primary palatoschisis (arrowhead), a midline cleft of the primary palate. These observations are consistent with craniofacial anomalies commonly associated with body wall complex malformations. Subfigures (**C**) and (**D**) show left lateral and left ventrolateral views, respectively. Both views show amniotic membrane adhesions (arrows) to the left auricular pavilion and along a circumferential line encasing the oronasal and mentonian regions. These observations are consistent with amniotic band syndrome (ABS) and suggest an early disturbance in embryonic development affecting the craniofacial region.

Craniofacial anomalies included bilateral cheiloschisis and primary palatoschisis (Figure 4A,B), as well as cranial amniotic adhesions (Figure 4C,D). No limb malformations were identified. This constellation of findings was consistent with both BSA Type VI and amniotic band sequence (ABS) as well as PC Class 1.

### 3.2. Case 2

**Figure 5:**
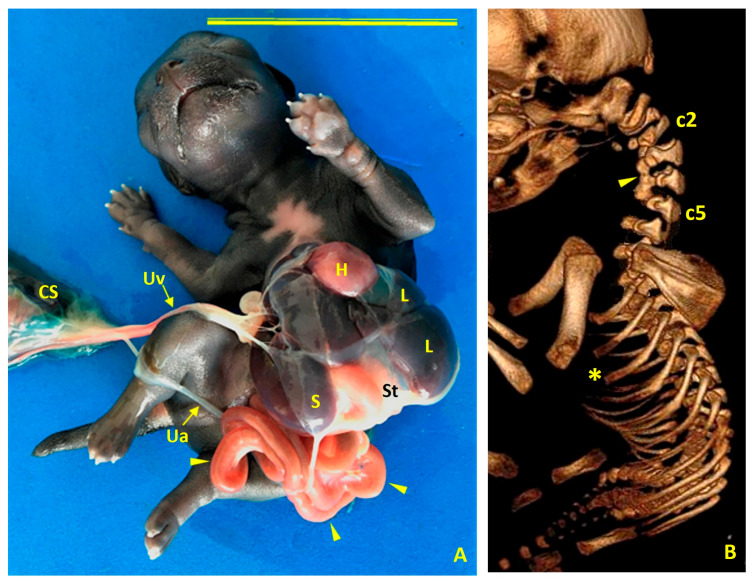
Case 2 (male Chihuahua) shows congenital body wall defects with ThAb. (**A**) Ventral view of the dog with lateral ThAb. The heart (H), liver (L), stomach (St), spleen (S), and intestinal loops (arrowheads) are exposed through the body wall defect into the extraembryonic coelomic cavity. Case 3 retains an intact umbilical cord (UC). CS: chorionic sac; Ua: umbilical artery; Uv: umbilical vein. Scale bar: 5 cm. (**B**) Computed tomography view of the thoracic and cervical malformations. This is a left lateral view of the dog showing complete sternal agenesis (asterisk), fusion of the cervical vertebrae C2-C5 (arrowhead), and an atlanto-occipital-axial malformation.

Case 2 was a male Chihuahua with thoracic aortic (ThAb) and an anomalous umbilical cord (UC) (Figure 5A). The dog was imaged using the Albira ARS II system (Bruker, Germany) at the Bioimagen Complutense (BIOMAC) Singular Scientific and Technical Facility (ICTS), with acquisition parameters of 600 mA and 45 kV to achieve a spatial resolution of 150 µm. Imaging revealed complete sternal agenesis and cervical vertebral fusion (Figure 5B).

**Figure 6:**
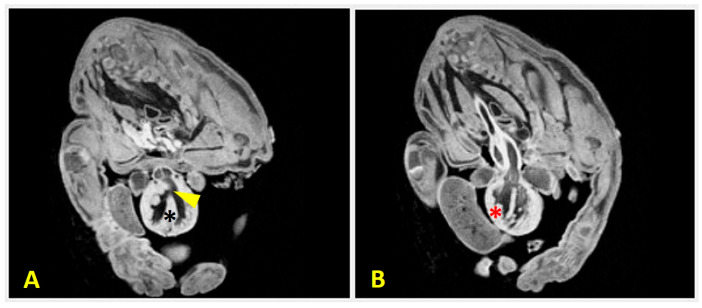
Magnetic resonance imaging (MRI) scans of Case 2 (male Chihuahua) showing congenital cardiac anomalies. (**A**) A transverse section showing a ventricular septal defect (arrowhead), which is identified as a discontinuity in the interventricular septum (black asterisk). This allows communication between the ventricles. (**B**) A transverse section showing right ventricular hypertrophy, evidenced by the thickening of the right ventricular wall (red asterisk). These results suggest complex cardiac malformations associated with body wall defects.

The dog also had ventral diaphragmatic agenesis and an absence of the pericardium. The heart exhibited ectopia cordis, a ventricular septal defect, and right ventricular hypertrophy (Figure 6).

An MRI provided a detailed visualization of the septal defect and myocardial thickening. Secondary palatoschisis and non-structural urogenital anomalies were present, but no limb malformations were observed. The case was consistent with BSA Type V, STBWC Type III, and PC Class 1.

### 3.3. Case 3

**Figure 7:**
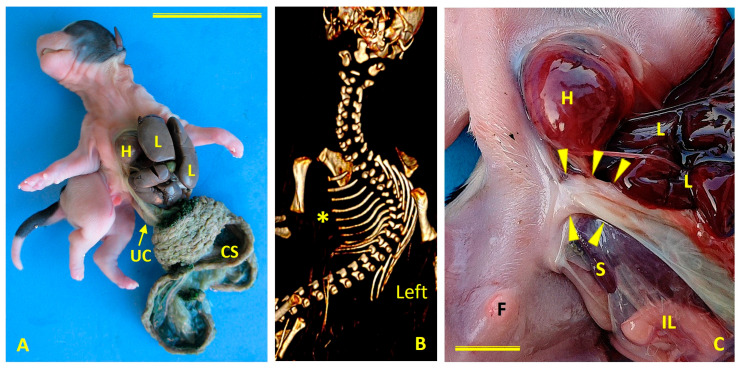
Congenital body wall defects in Case 3 (male Chihuahua) with lateral thoracoabdominoschisis (ThAb). (**A**) Ventral view of the externalized viscera. The dog retains an intact umbilical cord (UC). CS: chorionic sac; H: heart; L: liver. Scale bar: 5 cm. (**B**) Computed tomography (CT) scan showing thoracic and cervical malformations. This ventral view shows complete sternal agenesis (asterisk), right scoliosis, and hypoplastic left ribs, which are consistent with left thoracic wall hypoplasia. (**C**) Ventral view. The normal umbilical cord implantation is visible (arrows heads). The body wall defect is positioned laterally rather involving the midline. F, foreskin; H, heart; IL, intestinal loops; L, liver; S, spleen. Scale bar: 1 cm.

Case 3 was a male Chihuahua with lateral ThAb and a normally developed urinary catheter (UC) (Figure 7A). This dog was scanned using a Toshiba Aquilion 64 multislice CT scanner (Tokyo, Japan) with settings of 350 mA and 120 kV, a matrix of 512 × 512, and a field of view of 5.5 cm.

Structural anomalies included complete sternal agenesis, right-sided scoliosis, and hypoplastic left ribs (Figure 7B). The dog had normally developed UC positioned next to the lateral abdominal defect (Figure 7C). Cardiac findings included ectopia cordis and right ventricular hypertrophy. There were no craniofacial or limb anomalies, and non-structural urogenital defects were present. This phenotype aligned with pathogenetic classification class 2.

Cardiac anomalies in all cases were documented through gross dissection and MRI, with measurements included when feasible. Qualitative descriptions were used when quantitative assessment was not possible. Together, these three cases demonstrate the wide range of thoracoabdominal body wall defects and highlight the importance of considering morphological and pathogenetic criteria for proper classification.

### 3.4. General Phenotypic Overview

All three dogs presented with ThAb, which is characterized by a continuous defect involving both the thoracic and abdominal walls (Figure 2A, Figure 5A and Figure 7A). Two dogs exhibited central abdominal defects, and one displayed gastroschisis. Two cases displayed UC abnormalities, including shortened or dispersed umbilical vessels, which are consistent with BSA. In contrast, the third dog had a normally developed umbilical cord positioned adjacent to a lateral abdominal defect (Figure 7C). These variations highlight the phenotypic diversity of thoracoabdominal wall defects and their overlap with PC and BSA spectra. The phenotypic patterns documented in this study directly contributed to the development of a comparative anatomical classification of CS in humans and animals. A comparable classification was recently proposed from a One Health perspective, drawing in part on morphological insights consistent with those described in the present study [50].

## 4. Comparative Analysis

### 4.1. Comparative Diagnostic Analysis of All Cases

Table 2 summarizes the distinguishing features and underlying mechanisms of the three conditions identified in our case series. Of the seventeen canine cases reviewed, four exhibited overlapping features between PC and BSA, which complicated classification. All four cases showed ThAb, EC, and an anomalous UC—traits typically associated with PC—yet two of the cases also presented with limb or spinal anomalies characteristic of BSA. One case displayed craniofacial and limb constriction anomalies reminiscent of ABS. However, the overall presentation was more consistent with BSA. These findings support the existence of a phenotypic continuum between PC and BSA, particularly in incomplete forms. In contrast, Table 3 compiles cases reported in the literature where diagnostic discrepancies reflect the rarity and complexity of these conditions in veterinary medicine. Comparative patterns identified across our case series and published cases informed the recently proposed unified human–animal classification of Cantrell syndrome in veterinary sciences. This classification underscores the translational value of these canine cases within a one health framework.

Complex overlapping features were observed among the three studied canine cases, involving PC, BSA and ABS. Case 1 was classified as BSA Type VI with STBWC type III and PC Class 1, displaying features consistent with ABS. Case 2 was diagnosed as BSA Type V with SSBWC (sternal–spinal body wall complex) Type III and PC Class 1. Case 3, which presented with lateral Ab, was classified as PC Class 2. The bibliographic review included 17 additional cases, most of which were classified as PC Class 1, reflecting complete or near-complete manifestations of the syndrome. These included cases with intracardiac defects (cases 5–8), a sternal cleft (case 9), and a congenital peritoneal–pericardial hernia (cases 10–13). Cases 14, 16 and 20 were categorized as PC Class 2, demonstrating partial expression of the syndrome, which often involves peritoneopericardial diaphragmatic hernias. Case 15 was diagnosed as incomplete PC. Case 4 presented with a constellation of defects including cranioventral, abdominal wall, caudal, sternal, diaphragmatic, pericardial and intracardiac anomalies, supporting a PC Class 1 classification. Three cases (17, 19 and 1) were classified as BSA with varying STBWC types and concurrent PC features, highlighting the frequent overlap between these syndromes. Case 18 was diagnosed as omphalocele and interpreted by the authors as being part of the PC spectrum. These results demonstrate phenotypic and diagnostic overlap among PC, BSA, and ABS. This reinforces the importance of integrative frameworks that address their shared morphological features.

The bibliographic review revealed a wide range of body wall defects in the reported cases, reflecting the phenotypic variability of congenital thoracoabdominal defects. Cranioventral abdominal wall defects were the most frequently reported, appearing in five cases, and were often associated with complex syndromic presentations. Umbilical hernias were noted in three cases, while supraumbilical cutaneous atrophy was documented in one case, suggesting localized developmental disruption. One case presented a subxiphoid or ventral hernia, which was likely to contain hepatic parenchyma based on historical imaging and clinical description. Another case presented with a midline abdominal wall defect characterised by a large supraumbilical diastasis rectus. More severe disruptions included Ab in one case and ThAb in two cases, both of which are indicative of extensive failure of ventral body wall closure. The results highlight the similarities in the morphological features of these conditions and emphasize the importance of a structured approach to distinguishing between the major syndromic categories of body wall anomalies, particularly when classifying cases within the PC or BSA frameworks.

### 4.2. Comparative Patterns and Distinguishing Features of CS, BSA and ABS

Table 4 summarizes the key patterns identified across the three syndromes studied, highlighting their distinguishing features and underlying causes observed during the analysis.

A comparison of revised cases in the bibliography reveals consistent differences in diagnosis due to the condition’s complexity, their uncommon presentation in veterinary medicine and the presence of overlapping elements that make diagnosis challenging. This may inform future diagnostic or therapeutic approaches. The data was organized in this format to provide a concise visual reference that supports the interpretation of our findings and highlights the importance of syndrome-specific profiles in veterinary clinical practice.

## 5. Discussion

### 5.1. Overview of Body Wall Defects in a Clinical Context

Congenital body wall defects in dogs exhibit significant anatomical variability, which directly impacts diagnosis, prognosis, and clinical decision-making processes [2]. The cases examined in this study demonstrate the broad range of thoracoabdominal wall anomalies, from isolated abdominal defects to extensive thoracoabdominal (ThAb) involvement affecting both the thoracic and abdominal compartments. This diversity is similar to what has been reported in human medicine and emphasizes the necessity of flexible diagnostic frameworks in veterinary practice [64]. All three cases exhibited ThAb, confirming that this defect represents a severe disruption of ventral body wall formation. Two dogs presented with central abdominal defects and abnormal umbilical cords (UCs), and the third dog had gastroschisis with a normally developed UC. These patterns support the concept of a developmental continuum between Cantrell syndrome (CS) and body stalk anomaly (BSA), especially when UC abnormalities accompany extensive ventral wall disruption. A literature review further substantiated this continuum, revealing defects ranging from mild umbilical hernias to severe ThAb and complex syndromic presentations. Clinically, this underscores the importance of evaluating UC morphology, defect location, and visceral herniation when determining whether a case aligns with CS, BSA, or an overlapping phenotype.

### 5.2. Developmental Timing and Its Clinical Relevance

Understanding the embryonic timing of ventral body wall formation is essential for interpreting congenital defects in dogs. The primary body wall (PBW) forms between gestational days 20 and 23. Secondary body wall (SBW) fusion occurs between days 28 and 35, with final closure by days 40 and 45 [2]. Disruptions at different stages produce distinct clinical phenotypes. Case 1 exhibited thoracic arch (ThAb) malformation, a malformed umbilical cord (UC), and features consistent with amniotic band syndrome (ABS), which supports an early insult during PBW development. Early disruptions, characterized by mesodermal deficiency and persistence of the extraembryonic coelom, often result in widespread anomalies and poor prognosis [3,6]. Case 2 presented with a thoracolumbar hernia (ThAb), spinal anomalies, and a displaced umbilical cord (UC), suggesting a broader disturbance of the midline mesoderm during folding and segmentation. This pattern is consistent with amniotic band syndrome (ABS) Type V. In contrast, Case 3 presented with gastroschisis and a normal UC. This pattern is compatible with a later vascular disruption affecting the right omphalomesenteric artery or umbilical vein [64]. This type of localized defect typically arises after PBW and SBW fusion has begun. These distinctions are clinically meaningful. Early developmental insults tend to produce syndromic, multisystem anomalies, whereas later disruptions more often result in isolated defects with different prognostic implications. The Venn diagram developed in this study integrates developmental timing with anatomical findings, offering clinicians a tool to distinguish between syndromic and non-syndromic presentations.

### 5.3. EC: Anatomical Patterns and Diagnostic Implications

The cases of ectopia cordis (EC) reviewed were classified into subtypes based on characteristic combinations of sternal, abdominal, and umbilical cord (UC) anomalies. Type 2 EC was associated with omphalocele and abnormal UCs. Type 3 EC, on the other hand, involved a supraumbilical omphalocele and normal UCs. Case 3 represented type 4 EC, which combines a sternocostal defect with gastroschisis. These distinctions are clinically relevant because UC morphology and the nature of the abdominal wall defect help differentiate CS, BSA, and gastroschisis-associated EC. The observed phenotypic variability reinforces the need for a detailed anatomical assessment during necropsy or imaging because these features influence classification and potential surgical considerations [26].

### 5.4. Comparative Interpretation of CS, ABS, and BSA

Although CS, ABS, and BSA are traditionally described as distinct syndromes, the cases in this study demonstrate substantial overlap in their clinical and developmental features. All three conditions can involve ventral body wall defects, and limb or craniofacial anomalies may occur in ABS and BSA, occasionally mimicking CS. Case 1 exhibited amniotic bands consistent with ABS; however, the concurrent ThAb and UC abnormalities also met the criteria for BSA and CS. This finding is consistent with human and veterinary reports indicating a shared underlying pathway of early embryonic disruption in cases that fulfill multiple diagnostic criteria [1]. Recognizing this overlap is clinically important because it affects prognosis, recurrence risk, and communication with breeders or owners.

### 5.5. Diagnostic Overlap and the Syndromic Continuum

A clear pattern emerged across the 20 cases analyzed: many individuals exhibited features spanning more than one classification system [26,58,60,61]. ThAb, UC anomalies, and visceral herniation were prevalent in the CS, BSA, and ABS categories [26,60]. The simultaneous application of multiple classification systems in this study was intentional, reflecting the fact that rigid diagnostic boundaries often fail to capture the complexity of these anomalies. The findings support a syndromic continuum model, wherein CS, BSA, and ABS are overlapping outcomes of disruptions that occur at different developmental stages or through different mechanisms. While this continuum is presented as a comparative hypothesis rather than a definitive mechanistic conclusion, it offers clinicians a practical framework for evaluating complex congenital anomalies.

### 5.6. Clinical Features and Diagnostic Criteria in Practice

**Figure 8:**
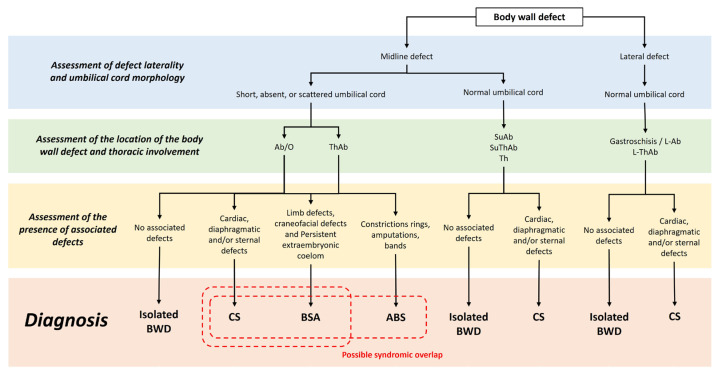
Clinical decision tree for evaluating and classifying congenital thoracoabdominal wall defects in dogs. Ab: abdominoschisis; L-Ab: lateral abdominoschisis; L-ThAb: lateral thoracoabdominoschisis; O: omphalocele; SuAb: supraumbilical abdominoschisis; SuThAb: supraumbilical thoraco-abdominoschisis; Th: thoracoschisis; and, ThAb: thoraco-abdominoschisis.

From a clinical perspective, an accurate diagnosis requires integrating anatomical findings with the developmental context. Congenital syringomyelia (CS) involves a defined pentalogy and is often accompanied by cardiac and diaphragmatic defects [4]. ABS is characterized by constriction rings, amputations, and asymmetric defects caused by fibrous bands [3,6,30,31,32,33,34,35,36,37,38]. BSA requires UC abnormalities, ThAb, and skeletal or sternal defects [1,2,3,5,6]. Several cases in this study met the criteria for more than one syndrome, which reinforces the need for clinicians to consider multiple diagnostic possibilities when evaluating neonates with extensive malformations [3]. UC morphology was a key discriminator between syndromic and non-syndromic presentations. To support clinical decision-making, Figure 8 summarizes the workflow for evaluating congenital thoracoabdominal defects in dogs. This tree summarizes the diagnostic workflow for congenital thoracoabdominal defects in dogs.

This algorithm uses defect location, thoracic involvement, UC morphology and associated structural anomalies to distinguish between CS, BSA, ABS, and non-syndromic defects, such as gastroschisis. This analytical model supports a practical, anatomically driven diagnostic approach that reflects the developmental continuum underlying these congenital anomalies. The decision tree is designed to help clinicians recognize the underlying syndromic patterns of these malformations, even when only partial clinical or imaging information is available. It accomplishes this by organizing defects according to their conserved embryologic and anatomical relationships.

To clarify its practical value, emphasis was placed on the fact that the clinical relevance of this system does not lie in the alteration of immediate therapeutic decisions. Rather, it lies in improving early syndromic recognition, guiding more consistent documentation and communication, and providing a structured approach that supports clinicians when diagnostic information is incomplete.

### 5.7. Pathogenesis, Etiology, and Evidence Gaps

The etiologies of CS, ABS, and BSA remain incompletely understood [4,51,65]. CS may involve early blastogenic disturbances with potential genetic contributions [4,65], whereas ABS and BSA are generally sporadic and linked to mechanical or vascular disruption [6]. The presence of amniotic bands in some BSA cases suggests partial pathogenetic overlap [3]. However, the evidence base in dogs is limited, with most conclusions relying on gross anatomical findings rather than molecular or genetic data. Future research integrating developmental biology, advanced imaging, and genomic approaches is needed to clarify the mechanisms underlying these syndromes.

### 5.8. Study Limitations

These conditions are rare in dogs, limiting sample size and the generalizability of findings. Reliance on published case reports introduces variability in diagnostic detail and potential publication bias. Heterogeneity in breeds, time periods, and clinical practices further complicates comparisons across cases. The absence of genetic or molecular data restricts mechanistic interpretation. Cross-species studies will be essential for refining classification systems and validating the proposed continuum model.

## 6. Conclusions

This study provides an integrated clinical, anatomical, and developmental analysis of congenital thoracoabdominal wall defects in dogs. It demonstrates that these anomalies often cannot be easily categorized. The three original cases, along with 17 cases reviewed from the literature, reveal significant phenotypic overlap among Cantrell syndrome (CS), amniotic band syndrome (ABS), and body stalk anomaly (BSA). Instead of being entirely distinct entities, these conditions appear to form a syndromic continuum shaped by the timing, severity, and mechanism of embryologic disruption. A key contribution of this work is emphasizing umbilical cord morphology, defect location, and thoracic involvement as practical diagnostic indicators. When interpreted within an embryologic framework, these features allow clinicians to distinguish syndromic from non-syndromic presentations more accurately. The diagnostic decision tree developed in this study provides a structured, anatomically grounded approach to support early recognition, consistent documentation, and clearer communication with breeders and owners. The findings also highlight critical gaps in current knowledge. Most veterinary diagnoses rely on gross morphology alone, and there is limited molecular or genetic data available to clarify pathogenesis. Future research integrating developmental biology, advanced imaging, and genomic analysis is essential for refining classification systems and validating the proposed continuum model. Overall, this study underscores the value of a developmental and comparative perspective in understanding congenital body wall defects. By reframing CS, ABS, and BSA as overlapping outcomes of related embryologic disturbances, the study provides a more flexible and clinically meaningful framework for evaluating these rare but significant anomalies in veterinary medicine.

## Figures and Tables

**Table 1:** Characteristics of dogs included in the study (study group).

Case	Sex	Weight (g)	LV-C (cm)	BWD (cm)	Birth Information					Breed
					Birth Information	Dam’s Age (Years)	Litter Size	GT (Days)	CM	
C1	F	665	19.3	7.3 × 3.1	Emergency C-section (BT)	4	2	65	AI	German Shepherd
C2	M	55	7.8	1.2 × 0.7	Emergency C-section (BT)	1,5	3	61	AI	Chihuahua
C3	M	110	10.9	3.5 × 1.2	Emergency C-section (BT)	3	3	62	NM	Chihuahua

AI: artificial insemination; BT: Birth type; BWD: Body wall defect; CM: Conception method; GT: Gestation time; LV-C: Length of vertex cauda; NM: Natural mating.

**Table 2:** Summary of defects evaluated and diagnosed in the case series.

Reference/Case		BWD	SpD	UCD	LD	StD	DD	PD	CD		CrfD	UGD	ABS	Proposed Diagnosis
C1	Case 1 ♀ German shepherd	ThAb	-	+	-	Short and cleft	AgVP	Ag	MVS, ASD, HLV, TVD	EC Type 2	BCh, PP	-	+	BSA TYPE VI STBWC III PC Class 1 ABS
C2	Case 2 ♂ Chihuahua	ThAb	VF, OAAD	+	-	Ag	AgVP	A	GH, VSD, RVH	EC Type 2	SP	NSt	-	BSA TYPE V SSBWC III PC Class 1
C3	Case 3 ♂ Chihuahua	L-ThAb	Sc	-	-	Ag	AgVP	A	RVH	EC Type 4	-	NSt	-	PC Class 2

A, absent; ABS, amniotic band syndrome; Ag, agenesis; AgVP, agenesis of the ventral portion; ASD, atrial septal defect; BCh, bilateral cheiloschisis; BWD, body wall defect; CrfD, craniofacial defect; DD, diaphragmatic defect; EC, *Ectopia cordis*; GH, globular heart; HLV, hypoplasia of the left ventricle; L-ThAb, lateral thoracoabdominoschisis; MVS, mitral valve stenosis; NSt, non-structural; OAAD, occipito-atlanto-axial defect; PD, pericardial defect; PP, primary palatoschisis; RVH, right ventricular hypertrophy; Sc, scoliosis; SP, secondary palatoschisis; SpD, spinal defect; StD, sternal defect; ThAb, thoracoabdominoschisis; TVD, tricuspid valve dysplasia; UCD, umbilical cord defect; UGD, urogenital defec; VF, vertebral fusion; and, VSD, ventricular septal defect.

**Table 3:** Summary of the congenital defects identified in the literature cases. It includes the original diagnoses by the cited authors and the proposed diagnostic classifications based on an integrated morphological and pathogenetic analysis.

Reference/Case		BWD	SpD	UCD	LD	StD	DD	PD	CD		CrfD	UGD	ABS	Author’s Diagnosis	Proposed Diagnosis
[55]	Case 4 ∅ G Cocker spaniel	Crv AWD	∅	-	-	Short and cleft	VS	CVD	ScThEC, VSD	EC Type 3	∅	∅	-	Congenital cranioventral abdominal wall, caudal sternal, diaphragmatic, pericardial, and intracardiac defects	PC Class 1
	Case 5 ∅ G Cocker spaniel	Crv AWD	∅	-	-	Short and cleft	VS	CVD	ScThEC, VSD	EC Type 3	∅	∅	-	Congenital cranioventral abdominal wall, caudal sternal, diaphragmatic, pericardial, and intracardiac defects	PC Class 1
	Case 6 ∅ G Cocker spaniel	Crv AWD	∅	-	-	Short and cleft	VS	CVD	ScThEC, VSD	EC Type 3	∅	∅	-	Congenital cranioventral abdominal wall, caudal sternal, diaphragmatic, pericardial, and intracardiac defects	PC Class 1
	Case 7 ∅ G Cocker spaniel	Crv AWD	∅	-	-	Short and cleft	VS	CVD	ScThEC	EC Type 3	∅	∅	-	Congenital cranioventral abdominal wall, caudal sternal, diaphragmatic, and pericardial defects	PC Class 1
	Case 8 ∅ G Cocker spaniel	Crv AWD	∅	-	-	Short and cleft	VS	CVD	ScThEC	EC Type 3	∅	∅	-	Congenital cranioventral abdominal wall, caudal sternal, diaphragmatic, and pericardial defects	PC Class 1
[56]	Case 9 ♂ German shepherd	SCA	∅	-	-	Cd cleft	Large VS	CVD	PDA, PLCVC	∅	∅	∅	-	Sternal Cleft Associated with Cantrell’s Pentalogy	PC Class 1
[57]	Case 10 ∅ G ∅ B	-	∅	-	-	UBCS	PPDH	PPDH	VSD, SaS	∅	∅	∅	-	PPDH	PC Class 1
	Case 11 ∅ G ∅ B	UH	∅	-	-	UBCS	PPDH	PPDH	VSD, SaS	∅	∅	∅	-	PPDH	PC Class 1
	Case 12 ∅ G ∅ B	-	∅	-	-	UBCS	PPDH	PPDH	VSD, SaS	∅	∅	∅	-	PPDH	PC Class 1
	Case 13 ∅ G	-	∅	-	-	UBCS	PPDH	PPDH	VSD, SaS	∅	∅	∅	-	PPDH	PC Class 1
[58]	Case 14 ♂ German shepherd	SXH	∅	-	-	Short	PPDH	Cr	∅	∅	∅	∅	-	PPDH	PC Class 2
[59]	Case 15 ♂ Border terrier	SU AWD	∅	-	-	Short and cleft	Ct	D	ScThEC	EC Type 3	∅	∅	-	Incomplete pentalogy of Cantrell	PC Class 1
[60]	Case 16 ♂ German shepherd	UH	∅	-	-	Short and cleft	PPDH	D	∅	∅	∅	∅	-	Unusual peritoneopericardial diaphragmatic hernia associated with a pericardial pseudocyst	PC Class 2
[61]	Case 17 ♂ Epagneul papillon	ThAb	∅	∅	PM	Ag	Hp	Ag	∅	EC Type 2	∅	NSt	-	Pentalogy of Cantrell	BSA TYPE V STLBWC III PC Class 1
	Case 18 ♂ Epagneul papillon	Ab	∅	∅	-	-	Hp	-	∅	NR	∅	∅	-	Pentalogy of Cantrell	Omphalocele
[26]	Case 19 ♂ German shepherd	ThAb	∅	∅	-	Ag	-	-		EC Type 2	∅	∅	-	Thoracic ectopia cordis, sternal agenesis, partial ectopia hepática and fissure abdominalis	BSA TYPE VI STBWC III PC Class 3
[62]	Case 20 ♀ Mixed breed	UH	∅	-	-	Cleft	Ct	PPDH	∅	∅	∅	∅	-	PPDH	PC Class 2

∅, not reported; ABS: amniotic band syndrome; Ag: agenesis; AWD: abdominal wall defect; BWD: body wall defect; Cd: caudal; Cr: cranial; CrfD: craniofacial defect; Crv: cranio-ventral; Ct: central; CVD: caudo-ventral defect; D: diaphragmatic; DD: diaphragmatic defect; EC, *Ectopia cordis;* G: gender; Hp: hipoplasia; NSt: non-structural; PD: pericardial defect; PDA: patent ductus arteriosus; PLCVC: persistent left cranial vena cava; PPDH: peritoneopericardial diaphragmatic hernia; SaS: subaortic stenosis; SCA: Supraumbilical cutaneous atrophy; ScThEC: subcutaneous thoracic *Ectopia cordis*; SpD: spinal defect; StD: sternal defect; SU: supraumbilical; SXH: Subxiphoid hernia; ThAb: thoracoabdominoschisis; TVD: tricuspid valve dysplasia; UBCS: underdeveloped bifurcated caudal sternebra; UCD: umbilical cord defect; UGD: genitourinary defect; UH: umbilical hernia; and, VS: v-shaped; VSD: ventricular septal defect.

**Table 4:** Comparative features and underlying causes of the three syndromes studied, including bibliographically reviewed and the three cases studied.

Syndrome	Comparative Features	Underlying Causes	References	Cases Studied
CS (Cantrell’s Syndrome)	Anomalous umbilical cord, midline-umbilical abdominal defect, cleft sternum, incomplete diaphragm and pericardium, *Ectopia cordis* and intracardiac anomalies.	Abnormal cephalic folding, possible chromosomal involvement.	[4,61,63]	C1, C2, C3, C4, C5, C6, C7, C8, C9, C10, C11, C12, C13, C14, C15, C16, C17, C19, C20
ABS (Amniotic Band Syndrome)	Constriction rings, limb amputations, craniofacial anomalies, body wall defects.	Amniotic membrane rupture, fibrous band entrapment, resulting in deformation, malformation or disruption.	[6,43]	C1
BSA (Body Stalk Anomaly)	Body wall defects, structural skeletal anomalies, anomalous umbilical cord, persistent extraembryonic coelom.	Faulty folding in all three embryonic axes.	[2,3,5]	C1, C2, C17, C19

## Data Availability

The original contributions presented in this study are included in the article. Further inquiries can be directed to the corresponding author.

## Abbreviations

The following abbreviations are used in this manuscript:

A	Absent
AB	Amniotic band
Ab	Abdominoschisis
ABS	Amniotic band syndrome
AI	Artificial insemination
ADC	Amniotic disruption complex
Ag	Agenesis
AgVP	Agenesis of the ventral portion
Ao	Aorta
ASD	Atrial septal defect
BCh	Bilateral cheiloschisis
BIOMAC	Biomagen Complutense
BSA	Body stalk anomaly
BT	Birth type
BrT	Brachiocephalic trunk
BWD	Body wall defect
Cd	Caudal
cdVC	Caudal vena cava
CM	Conception method
Cr	Cranial
CrfD	Craniofacial defect
CrvAWD	Cranioventral abdominal wall defect
CrVC	Cranial vena cava
CS	Cantrell Syndrome
Ct	Central
CT	Computed Tomography
CVD	Cranio-ventral defect
D	Diaphragmatic
DD	Diaphragmatic defect
EC	*Ectopia cordis*
F	Foreskin
GA	Gallbladder agenesis
GH	Globular heart
GT	Gestation time
GuD	Genitourinary defect
H	Heart
HLV	Hypoplasia of the left ventricle
Hp	Hypoplasia
HR	Hypoplastic ribs
ICTS	Singular Scientific and Technical Facility
IL	Intestinal loops
LBWC	Limb body wall complex
L	Liver
lSA	Left subclavian artery
L-ThAb	Lateral thoracoabdominoschisis
lV	Left ventricle
LV-C	Length of vertex cauda
MRI	Magnetic resonance imaging
MVS	Mitral valve stenosis
NM	Natural mating
NS-B	Breed not specified by authors
NS-G	Gender not specified by authors
NSt	Non-structural
OAAD	Occipito-atlanto-axial defect
OD	Other defects
Omph	Omphalocele
P	Pancreas
PC	Pentalogy of Cantrell
PD	Pericardial defect
PDA	Patent ductus arteriosus
PLCVC	Persistent left cranial vena cava
PP	Primary palatoschisis
PPDH	Peritoneopericardial diaphragmatic hernia
PS	Pulmonary stenosis
rA	Right auricle
rAt	Right atrium
rV	Right ventricle
RVH	Right ventricular hypertrophy
S	Spleen
SaS	Subaortic stenosis
Sc	Scoliosis
SCA	Supraumbilical cutaneous atrophy
ScThEC	Subcutaneous thoracic ectopia cordis
SP	Secondary palatoschisis
SPBWC	Spinal body wall complex
SPLBWC	Spinal limb body wall complex
SSBWC	Sternal spinal body wall complex
St	Stomach
STBWC	Sternal body wall complex
STLBWC	Sternal limb body wall complex
SpD	Spinal defect
St	Structural
StD	Sternal defect
SUA	Single umbilical artery
SUAWD	Supraumbilical abdominal wall defect
SV	Single ventricle
SXH	Subxiphoid hernia
ThAb	Thoracoabdominoschisis
TVD	Tricuspid valve dysplasia
Ua	Umbilical artery
UBCS	Underdeveloped bifurcated caudal sternebra
UC	Umbilical cord
UCD	Umbilical cord defect
ud	Undescribed
UH	Umbilical hernia
Uv	Umbilical vein
VS	V-shaped
VSD	Ventricular septal defect
